# Role for the Unfolded Protein Response in Heart Disease and Cardiac Arrhythmias

**DOI:** 10.3390/ijms17010052

**Published:** 2015-12-31

**Authors:** Man Liu, Samuel C. Dudley

**Affiliations:** The Warren Alpert Medical School of Brown University, Lifespan Cardiovascular Institute, the Providence VA Medical Center, 593 Eddy Street, APC814, Providence, RI 02903, USA; man_liu@brown.edu

**Keywords:** PERK, IRE1, ATF6α, Grp78, cardiac ion channels, ischemia

## Abstract

The unfolded protein response (UPR) has been extensively investigated in neurological diseases and diabetes, while its function in heart disease is less well understood. Activated UPR participates in multiple cardiac conditions and can either protect or impair heart function. Recently, the UPR has been found to play a role in arrhythmogenesis during human heart failure by affecting cardiac ion channels expression, and blocking UPR has an antiarrhythmic effect. This review will discuss the rationale for and challenges to targeting UPR in heart disease for treatment of arrhythmias.

## 1. Introduction

The endoplasmic reticulum (ER) is a cellular organelle that forms an interconnected space for secreted and membrane protein translation, folding and assembling before trafficking in vesicles to the Golgi apparatus, cytosol and plasma membrane. Only properly folded proteins can be transported out of the ER. Three major characteristics of the ER lumen ensure correct protein folding: the highly oxidative environment that favors the formation of disulfide bonds to convey the tertiary and quaternary structure of proteins, the highly expressed chaperone proteins to guild protein folding, and the high Ca^2+^ concentration essential for Ca^2+^-dependent chaperones-protein interactions. Any disturbance of these three determinants can lead to protein unfolding or misfolding. Unfolded or misfolded proteins accumulate in the ER lumen, causing ER stress and triggering of the unfolded protein response (UPR). 

The UPR is a stress response highly conserved in evolution from yeast to all mammalians. Short-term ER stress-induced UPR is adaptive and plays protective roles for cell survival by eliminating misfolded proteins. Nevertheless, when the ER stress is prolonged, the UPR can lead to cell dysfunction and eventually cell death. Unfolded protein accumulation triggers glucose regulated protein-78 (Grp78) to dissociate from three UPR sensors. These sensors are the double stranded RNA-activated protein kinase-like ER kinase (PERK), inositol-requiring ER-to-nucleus signal kinase 1 (IRE1), and activating transcription factor 6α (ATF6α). Grp78 dissociation triggers oligomerization and auto-phosphorylation of the UPR sensors and leads to their activation. Meanwhile, Grp78 binds to misfolded or unfolded proteins as a chaperone to help them refold correctly or guide them for the ER-associated degradation (ERAD). The activated UPR sensors initiate three branches of signaling transduction, leading to downregulation of protein translation, generation of chaperone proteins, and expression of genes and proteins that restore the protein folding capacity in the ER. The chaperones including Grp78, Grp94, calnexin, and calreticulin [1] play essential roles in the ERAD process by binding to misfolded or unfolded proteins and helping them refold or exit the ER to be degrading in the cytosol. These UPR sensors and chaperones have been used as ER stress markers. 

**Figure 1:**
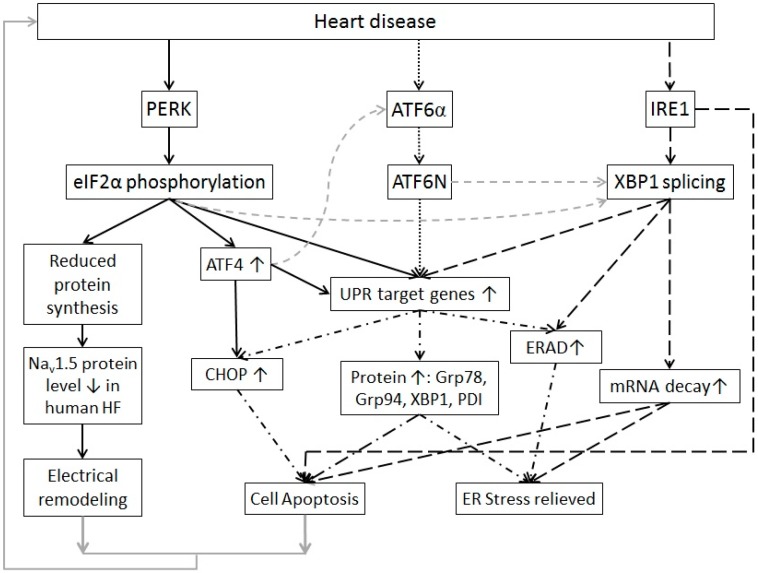
A scheme of signaling pathways of the unfolded protein response with its sensors, downstream effectors, targeting genes, and possible outcomes. The PERK branch (linked by black-solid arrows) can be activated in human heart failure, diabetes hearts and ischemia/reperfusion, leading to inhibition of nascent protein synthesis and ATF4 elevation [10,11,12,13]. Elevated ATF4 can induce expression of CHOP and other UPR target genes. The ATF6 branch (linked by black-dotted arrows) is reported to be activated in early stage of ischemia and mainly induces UPR target genes expression [14,15,16]. The IRE1 branch (linked by black-dashed arrows) is reported to be activated in human heart failure and ischemia/reperfusion [12,17,18]. Its downstream effectors activate the ERAD and ER-localized mRNA decay [5]. The IRE1 branch has also been reported to promote cell apoptosis via Jun N-terminal kinase and p38 MAPK [19]. All three branches cause UPR target genes expression (linked by dash dotted dash arrows) such as the UPR chaperones Grp78 and Grp94 and ERAD proteins to help misfolded/unfolded protein refold or degrade. There is also crosstalk among the three branches (linked by gray dashed arrows). Under short-term and mild ER stress, the UPR is adaptive to release the ER stress; while under prolonged and severe ER stress, the UPR leads to cell apoptosis and electrical remodeling (due to decreased cardiac ion channels) that will harm the cardiac function and lead to heart diseases (linked by gray arrows).

The three UPR branches (Figure 1) have distinct signaling pathways either to enhance protein folding ability or to attenuate protein synthesis and loading of the ER. For the PERK branch, phosphorylated translation initiation factor 2α (eIF2α) and ATF4 are the direct downstream effectors. Activation of the IRE1 branch leads to elevated spliced X-box binding protein 1 (sXBP1). For the ATF6α branch, the cleaved N-terminus of ATF6α (ATF6N) is the downstream effector. All three branches induce elevated expression of the UPR chaperones, but each branch appears to have unique roles as well. The PERK branch attenuates global protein synthesis and acts to preserve redox balance during ER stress through activation of ATF4 and NRF2, respectively [2,3]. The IRE1 branch effector sXBP1 regulates lipid biosynthetic enzymes and ERAD proteins [4]. It is also reported to induce decay of ER-localized mRNAs [3,5]. The ATF6α branch plays a major role in promoting the UPR target genes expression. Crosstalk among the three branches exists. For instance, eIF2α phosphorylation can activate NF-κB [6,7], which induces downregulation of sXBP1 mRNA levels via microRNA-30c-2* [8]. ATF4 can activate the ATF6α branch through enhancing ATF6α expression and translocation to the Golgi [9]. This interplay may make the UPR more redundant and more complicated to target pharmacologically. 

Cardiomyocytes are the fundamental units that make up the cardiac muscle and perform its contractile function. In adult cardiomyocytes, being terminally differentiated and lacking significant regenerative potential, protein quality control is crucial for cardiomyocyte survival and function. The UPR is one of the important mechanisms of protein quality control. The sarco/endoplasmic reticulum (SER) of cardiomyocytes is not only the place where membrane proteins such as cardiac ion channels undergo synthesis, folding, and assembling, but is also the Ca^2+^ reservoir for a normal excitation-contractility coupling. It is therefore critical for both general cellular function and myocyte contractility. Disturbance and stress of the SER is common in heart disease and can activate the UPR. 

The UPR has been investigated extensively in neurodegenerative diseases and diabetes but not in heart disease. In the past decade, the UPR has been found to play important roles in pathological cardiac hypertrophy, dilated cardiomyopathy, ischemic cardiomyopathy, diabetic heart disease, and human heart failure [10,15,17,20,21,22]. In this review, we will discuss which UPR modulators and effectors are involved in heart diseases and how this understanding may point the direction for future therapeutics by modulating the UPR activation. 

## 2. The UPR in Heart Disease

In heart disease, the ER is often under stress because of oxidative stress, hypoxia, and glucose deprivation. Activated UPR leads to suppression of protein translation, and seems to be responsible for downregulation of cardiac ion channels [10]. Disturbed Ca^2+^ homeostasis can alter the Ca^2+^ transient and cardiomyocyte contraction. Altered protein homeostasis can perpetuate cardiomyocyte dysfunction.

### 2.1. UPR in Cardiac Hypertrophy

Activation of the UPR has been observed in cardiac hypertrophy, a condition associated with arrhythmic risk. Elevated CHOP, a downstream pro-apoptotic effector of the PERK branch of the UPR (Figure 1), has been observed in a mouse model of pressure-overload induced cardiac hypertrophy and is in part responsible for increased apoptosis and deterioration of contractility that characterize longstanding hypertrophy [23,24]. ASK1, downstream in the IRE1 branch, is crucial for cell death induced by the ER stress. Knockout of ASK1 reduces cardiac dysfunction and cell apoptosis in pressure-overload induced cardiac hypertrophy [25]. Activation of ATF6 is also observed in cardiac hypertrophy [26]. Its upregulation seems to play a protective role. 

### 2.2. UPR in Ischemia

All three UPR branches are activated in ischemia. Under prolonged ischemia or hypoxia, Grp78, XBP1, ATF4, eIF2α, and CHOP are elevated at the mRNA and protein levels in cardiomyocytes, indicating activation of the IRE1 and PERK branches of the UPR [12,17]. A downstream effector of the PERK branch, Tribbles 3 (an intracellular pseudokinase), is significantly elevated in myocardial infarction [11,14]. Transgenic mice with cardiac-specific overexpression of Tribbles 3 show pathological cardiac remodeling after myocardial infarction [14]. An ER stress response gene downstream of PERK, p53-upregulated modulator of apoptosis (Puma), induces cell apoptosis when overexpressed in cardiomyocytes [27]. Puma knockout shows protective effects during ischemia/reperfusion *in vivo* [27]. These results suggest that Puma plays a detrimental role in ischemia/reperfusion, and inhibition of Puma may be beneficial for myocardial infarction and heart failure. The ATF6α branch is activated in ischemia and inactivated upon reperfusion [15], suggesting that the ATF6α branch plays an inducible role during ischemia that may affect preconditioning during reperfusion. ATF6α activation has been found to show protect effects during ischemia/reperfusion in a transgenic mouse model with cardiac-restricted expression of activated ATF6 [28]. 

### 2.3. UPR in Heart Failure

The PERK and IRE1 branches of the UPR are activated in human failing hearts. These hearts show increased mRNA or protein levels of Grp78, sXBP1, PERK, ATF4, and CHOP [10,18]. Elevated Grp78 and CHOP have been reported in mouse models of heart failure [23,29]. Recently, our group reported activated UPR in human failing heart tissue with elevated PERK, CHOP and calreticulin [10]. Elevated ATF6 protein levels (although not the activated form ATF6N) have been reported in human heart failure patients with dilated and ischemic cardiomyopathy [30].

A hallmark of heart failure is aberrant Ca^2+^ homeostasis. SER Ca^2+^ ATPase isoform 2a (SERCA2a) and 3f (SERCA3f) are both altered in heart failure. SERCA3f is upregulated in human failing hearts, and overexpression of this Ca^2+^ pump increases XBP1 splicing and Grp78 expression [31]. This indicates that SERCA3f is likely an UPR effector and participates in the ER stress in human heart failure. PERK helps to maintain SERCA2a levels in a transaortic constriction model of heart failure, a potentially salutary effect [32]. 

### 2.4. UPR in Other Heart Diseases

ER stress and activated UPR have been reported in diabetic cardiomyopathy (reviewed in [21,33]). Activated UPR disturbs Ca^2+^ handling and increases oxidative stress and cell apoptosis, all of which contribute to the pathogenesis of diabetic cardiomyopathy [23]. Upregulation of several UPR effectors including Grp78, phosphor-eIF2α, ATF6N, CHOP, and cleaved caspase-12 is observed in streptozotocin-induced diabetic mouse and rat heart tissues [34,35], indicating the involvement of the PERK and ATF6α branches. Alcoholic cardiomyopathy may involve the UPR. ER stress can be induced by chronic alcohol ingestion. Ethanol elevates Grp78 and enhances activation of the PERK and IRE1 branches [36]. Cancer chemotherapy-induced cardiotoxicity is associated with activated PERK and IRE1 branches of UPR [37]. ER stress occurs in autoimmune cardiomyopathy with elevated Grp78, CHOP, and nuclear translocation of cleaved ATF6N [38]. Obesity-induced cardiomyopathy has been reported with increased Grp78 and activated PERK branch [39]. Patients with Huntington’s disease present a high incidence of cardiovascular events, likely resulted from accumulation of toxic amyloid-like inclusions. A study of cardiac amyloidosis model of *Drosophila* shows unfolded protein accumulation (poly-glutamine repeats that can cause aggregation-prone amyloidosis) and oxidative stress [40]. 

Human and mouse heart failure tissues exhibit high levels of soluble preamyloid oligomers, which are accumulation of misfolded protein monomers and dimers, and can cause toxicity in many neurodegenerative diseases. Transgenic mice with overexpression of long polyglutamine repeats exhibit accumulation of preamyloid oligomers, which leads to cardiomyocyte death and heart failure [41,42]. Notably, ER stress marker proteins such as Grp78, PERK, IRE1/XBP1, and ATF6, are not increased in this transgenic mice [42], although they have been reported to be elevated in some other aggregate-forming diseases such as hereditary hemochromatosis and spinocerebellar ataxia type 14 [43,44]. This indicates that a different mechanism other than the activation of the UPR regulates the cardiomyocyte death and heart failure in the transgenic mice. Little work has been done to investigate whether and how the UPR contributes to cardiovascular amyloidosis.

## 3. UPR and Arrhythmias

Heart failure is characterized by arrhythmogenic electrical remodeling consisting mostly of ion channel downregulations. Recently, our group found that PERK activation downregulates cardiac Na^+^ channel (Na_v_1.5) and cardiac rapidly activated K^+^ channel (K_v_4.3) in human heart failure [10]. The α subunit of cardiac Na_v_1.5 is encoded by *SCN5A*. Abnormal splicing of *SCN5A* is elevated in human heart failure and results in truncated mRNA variants, which are translated into nonfunctional channel proteins and trapped in the ER. This leads to PERK activation and causes downregulation of the full length normal Na_v_1.5 protein expression [10]. The reduction of full-length, functional Na_v_1.5 leads to a decreased Na^+^ current density and consequently decreased conduction velocity (Figure 1: the PERK branch) [15,45]. The effect of activated PERK is not specific to Na_v_1.5. UPR activation also results in a reduction of *KCND3* that encodes the α subunit of K_v_4.3. Cardiac K_v_4.3 channel conducts K^+^ current I_to_, which is the main contributor to the notch of phase 1 of the cardiac action potential and is responsible for early repolarization. Reduced I_to_ can increase membrane resistance, causing shortening of the cardiac action potential duration and phase 2 reentry [46,47]. Blocking PERK prevented these ion channel downregulations. Other ion channel downregulations in heart failure may also be the result of UPR. Therefore, inhibiting the UPR may reverse arrhythmogenic channel downregulation, representing a new paradigm of maintaining ion channel activity to prevent arrhythmia. 

Other mechanisms of arrhythmia under ER stress include alerted Ca^2+^ homeostasis. Under prolonged ER stress, Ca^2+^ release from the ER is increased [19], which can not only activate signaling pathways of cell apoptosis but also induce delayed afterdepolarizations that can cause cardiac arrhythmias. Another altered mechanism is ER-dependent ion channel glycosylation. A recent study shows that only a fully glycosylated cardiac Na_v_1.5 is trafficked normally [48]. Therefore, altered glycosylation during ER stress may contribute to ion channel alterations and arrhythmic risk.

## 4. Targeting the UPR in Heart Disease

### 4.1. Potential Benefits of UPR Inhibition

Prolonged UPR activation may contribute to the transition from compensated hypertrophy to dilated cardiomyopathy. Also, UPR-associated apoptosis could be a cause of continued cardiac decline in heart failure. UPR seems to be involved in ion channel downregulation that contributes to arrhythmic risk in heart disease. Therefore, inhibiting UPR may have several salutary benefits in heart disease to prevent contractile decompensation and increased arrhythmic risk.

### 4.2. Potential Methods to Inhibit the UPR

The benefit of UPR inhibition may depend on the method. One strategy to inhibit UPR is to prevent its activation by upstream signals. Oxidative stress appears to be an element of a positive feedback loop to activate the UPR [49,50]. Antioxidants such as thiols and butylated hydroxyanisole have been reported to attenuate the UPR in a bleeding disorder study [51]. Other possible strategies to inhibit the UPR include decreasing unfolded proteins, preventing the UPR sensors from activating, or inhibiting the activated UPR sensors and downstream effectors. In the case of the abnormal *SCN5A* mRNA splicing in heart failure, inhibiting the pathogenic splicing factors RBM25 and hLuc7A [52] reduces ER stress. To prevent activation of UPR sensors, Grp78 overexpression may be useful, which can enhance Grp78 binding to the UPR sensors. Moreover, Grp78 overexpression can increase the binding of Grp78 to unfolded/misfolded proteins to accelerate ERAD, which would further alleviate ER stress. Overexpression of Grp78 and Grp94 has been reported to attenuate hypoxia-mediated cardiomyocyte death [53] and protect cardiomyocytes against oxidative stress [54]. Chemical chaperones, such as 4-phenylbutyric acid (4-PBA) and taurine-conjugated ursodeoxycholic acid (TUDCA) have been reported to reduce the ER stress by preventing misfolded protein aggregation and alleviating ER stress (reviewed in [55,56]), mimicking the functions of ER chaperones. These chemical chaperones have been found to inhibit the elevation of phospho-eIF2α, ATF4, and CHOP in hypertension, leading to reduced cardiac damage and improved vascular function in hypertension [57]. Myocardial infarction in mice induces ER stress and provokes cardiac apoptosis and fibrosis, causing remodeling and cardiac rupture [58]. Treatment with 4-PBA decreases the protein levels of the ER stress markers and pro-apoptotic proteins, leading to prevention of these adverse outcomes [58]. Analogues of 4-PBA such as 2-POAA-OMe, 2-POAA-NO_2_, and 2-NOAA have been found to suppress the induction of Grp78 and CHOP and to inhibit the IRE1 and ATF6α pathways [59]. Therefore, 4-PBA and its analogues may be used to target the ER stress to prevent post-MI complication. TUDCA inhibits the elevation of Grp78, phospho-PERK and phospho-eIF2α in obese mice, leading to alleviation of obesity-induced cardiac hypertrophy, compromised fractional shortening and cardiomyocyte contractile [39]. TUDCA and 4-PBA also prevent aggregate formation by reducing ER stress [43]. TUDAC hinders the activation of the UPR, whereas 4-PBA enhances the degradation of misfolded proteins. 

Human anti-PERK short hairpin RNAmir has been used *in vitro* to block PERK activation in human induced pluripotent stem cell-derived cardiomyocytes [10]. Recently, GSK2606414, a small molecule oral agent, has been found to halt brain cell death by blocking PERK phosphorylation *in vivo* and has shown efficacy in prion-mediated disease associated with aggregation of misfolded protein in the ER [60]. This compound has been proposed as a new treatment for Alzheimer’s disease [61] and may be useful in heart disease. A similar compound GSK2656157 is reported to selectively inhibit PERK with antitumor and antiangiogenic activity [62]. An inhibitor of IRE1, 4μ8C, can block substrate access to the active site of IRE1 and selectively inactivate both XBP1 splicing and IRE1-mediated mRNA degradation [63]. This compound does not alter the cell response to the ER stress; instead, it reduces ER volume. This may limit its use in regulation of the ER stress. There is no specific inhibitor reported for the ATF6 branch to date [64].

## 5. Precautions on Targeting the UPR 

Whenever inhibiting a process like the UPR with salutary and deleterious effects, caution must be exercised. The UPR has shown protective effects in certain circumstances [17,28,53,54]. Therefore, timing and specific targeting of the UPR branches may be critical. For instance, activation of the ATF6α branch before ischemia reduces myocardial tissue damage during ischemia/reperfusion [28]. Inhibition of the PERK branch with apelin-13 protects the heart from ischemia-induced ER stress [65]. CHOP knockout mice show less cardiac hypertrophy, fibrosis and cardiac dysfunction compared with the wild type mice with transverse aortic construction [24]. Mice with inducible cardiac-specific PERK knockout have been shown to have normal heart function and not suffer from diabetes [32]. Nevertheless, the mouse heart ejection fraction is slightly decreased in response to chronic transverse aortic constriction when compared to control mice [32]. These studies indicate that inhibition of the PERK branch in ischemia and cardiac hypertrophy may be beneficial although complication may occur in states of pressure overload.

Homozygous knockout animal models of the UPR sensors and effectors have shown potentially harmful effects of inhibiting the UPR (reviewed in [66]). Elements of UPR are involved in development. Global knockout of XBP1 or IRE1α is embryonic lethal with incomplete development of the heart and blood vessels [67,68]. Global PERK knockout leads to diabetes mellitus and growth retardation in mice [69]. ATF6α knockout induces liver steatosis, hypoglycemia, and insulin resistance [70,71]. Therefore, UPR inhibition may not be suitable in pregnancy or childhood. Partial or temporary inhibition of the UPR sensors or organ-specific gene therapy may be the safest alternative.

## 6. Conclusions

Heart disease remains to be the most common cause of death in the developed countries. There is evidence showing that the UPR plays important roles in heart disease and modulating the UPR may be clinically beneficial. For example, in heart failure, UPR activation contributes to arrhythmic risk and inhibiting the UPR may decrease that risk. Nevertheless, the UPR can have salutary effects, and complete, whole body, or indefinite inhibition of even a single UPR branch may have untoward side effects. Further investigation and understanding of the UPR will help overcome these limitations and discover more precisely targeting agents or time limited treatment approaches. Overall, targeting the UPR seems to be a potentially fruitful approach in novel therapeutics for cardiac disease.
